# Supplementary material for: Polymerized β-Cyclodextrin-Based Injectable Hydrogel for Sustained Release of 5-Fluorouracil/Methotrexate Mixture in Breast Cancer Management: In Vitro and In Vivo Analytical Validations
Source: Pharmaceutics. 2022 Apr 8;14(4):817. doi: 10.3390/pharmaceutics14040817 (PMC9025605; doi:10.3390/pharmaceutics14040817)
Supplement: Supplementary file 1 [file pharmaceutics-14-00817-s001.zip › pharmaceutics-1623236-supplementary.pdf]

Polymerized β-cyclodextrin-based injectable hydrogel for sustained release of 5-fluorouracil/methotrexate mixture in breast cancer management. *In vitro* and *In vivo* Analytical Validations

1. SI. 1_Figure S1 (A and B)

A)

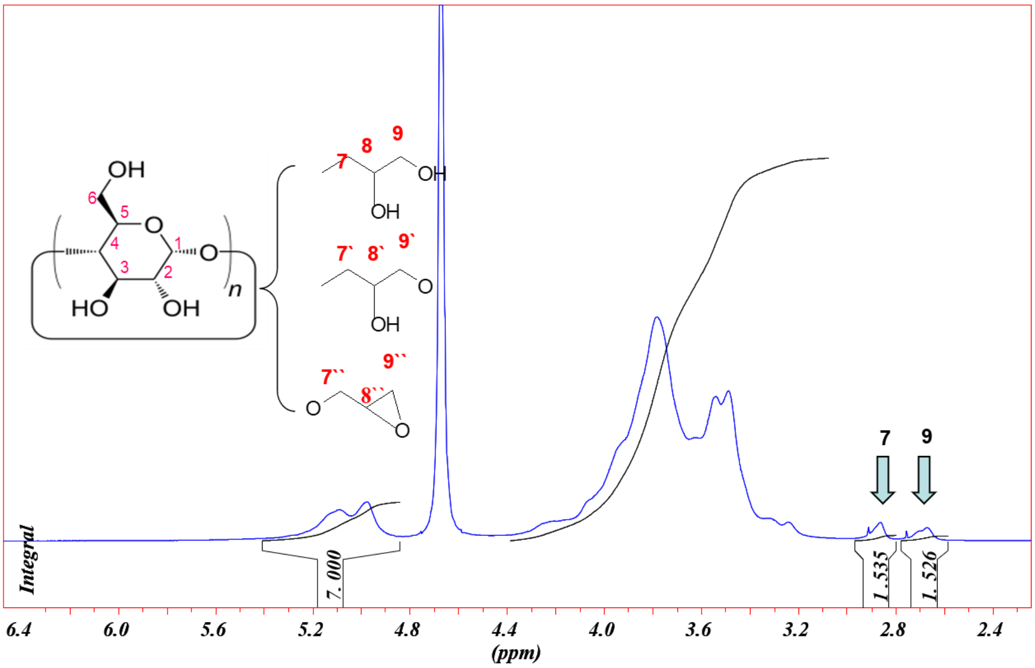

B)

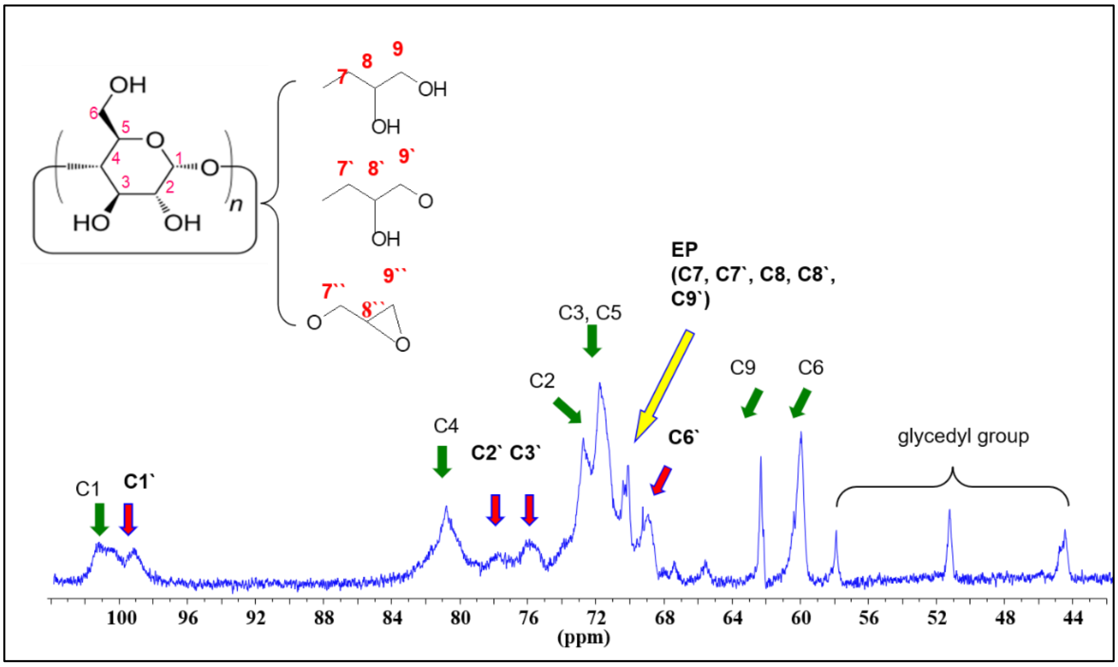

Figure S1. ^{1}H NMR (600 MHz, D_2O) spectrum of pβ-CD, (A). ^{13}C NMR (600 MHz, D_2O) spectrum of pβ-CD, (B).

2. SI. 2_Figure S2

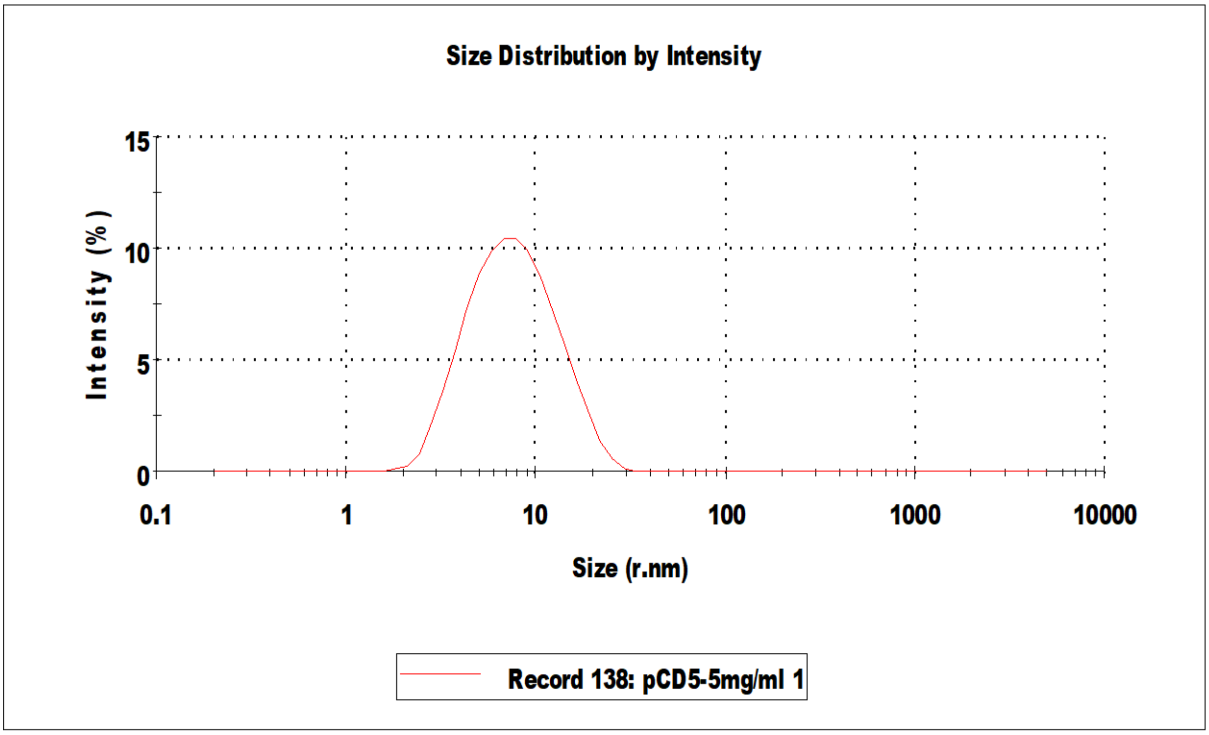

Figure S2. DLS spectrum of linear water soluble pβ-CD polymer synthesized from the interaction between10 g (8.8 mmole) β-CD and 5g (5.4 mole % or 54 mmole) epichlorohydrin in alkaline medium in the presence of toluene to produce linear polymer.

Condition of experiment:

Standard: ply(styrene) of RI 1.59,   Dispersant: water,   Temp: 25,   Attenuator: 9

Reading:

Size average (r.nm): 6.59   hydrodynamic diameter (d.nm): **8.22**   % intensity : 100

PI: 0,2

Mn (g/mol): on the light of hydrodynamic diameter and regarding the published data, the molecular weight of this polymer is 106 kD.

3. SI. 3._Figure S3

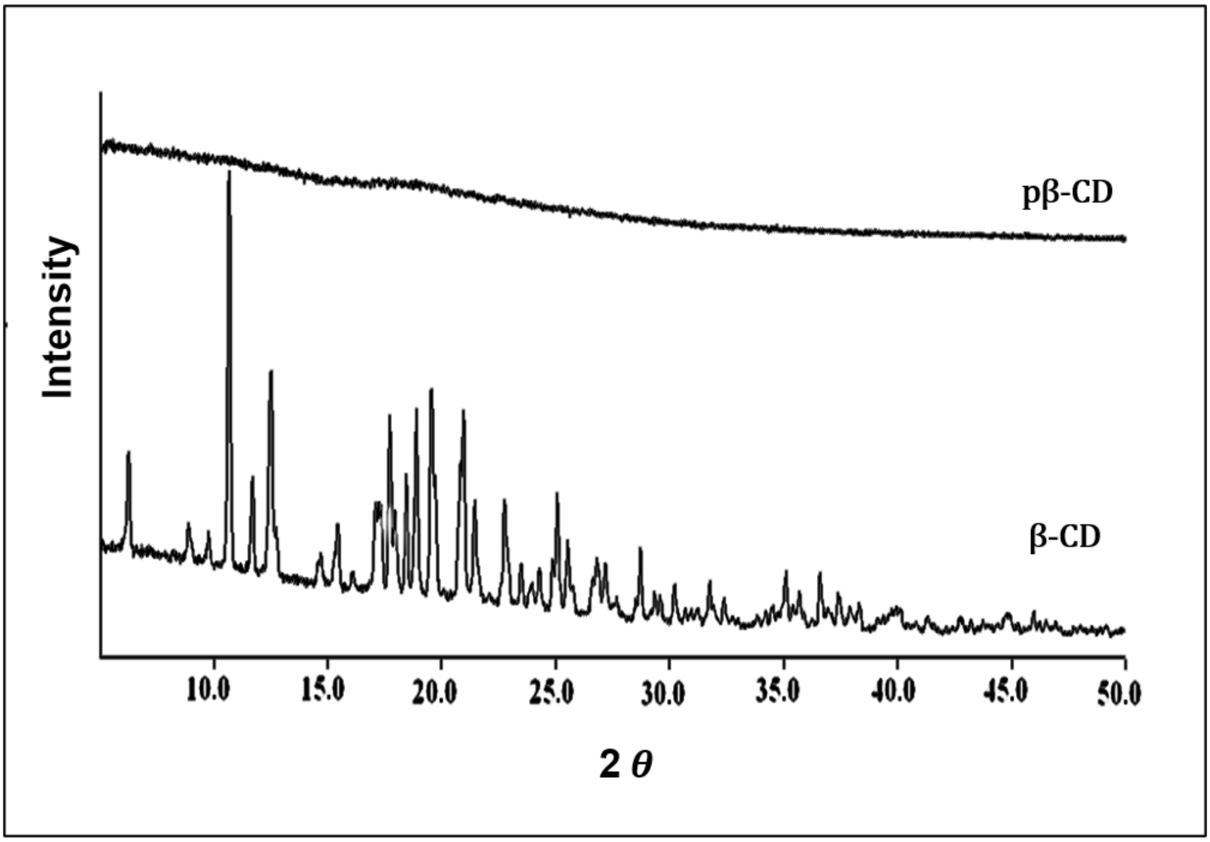

Figure S3. X-ray diffraction patterns of β-CD and pβ-CD, showing the complete disappearance of crystalline intensity peaks of native cyclodextrin upon polymerization. The x-ray confirmed the conversion of crystalline B-CD from crystalline form to amorphous one.

4. SI. 4_Figure S4 (A and B)

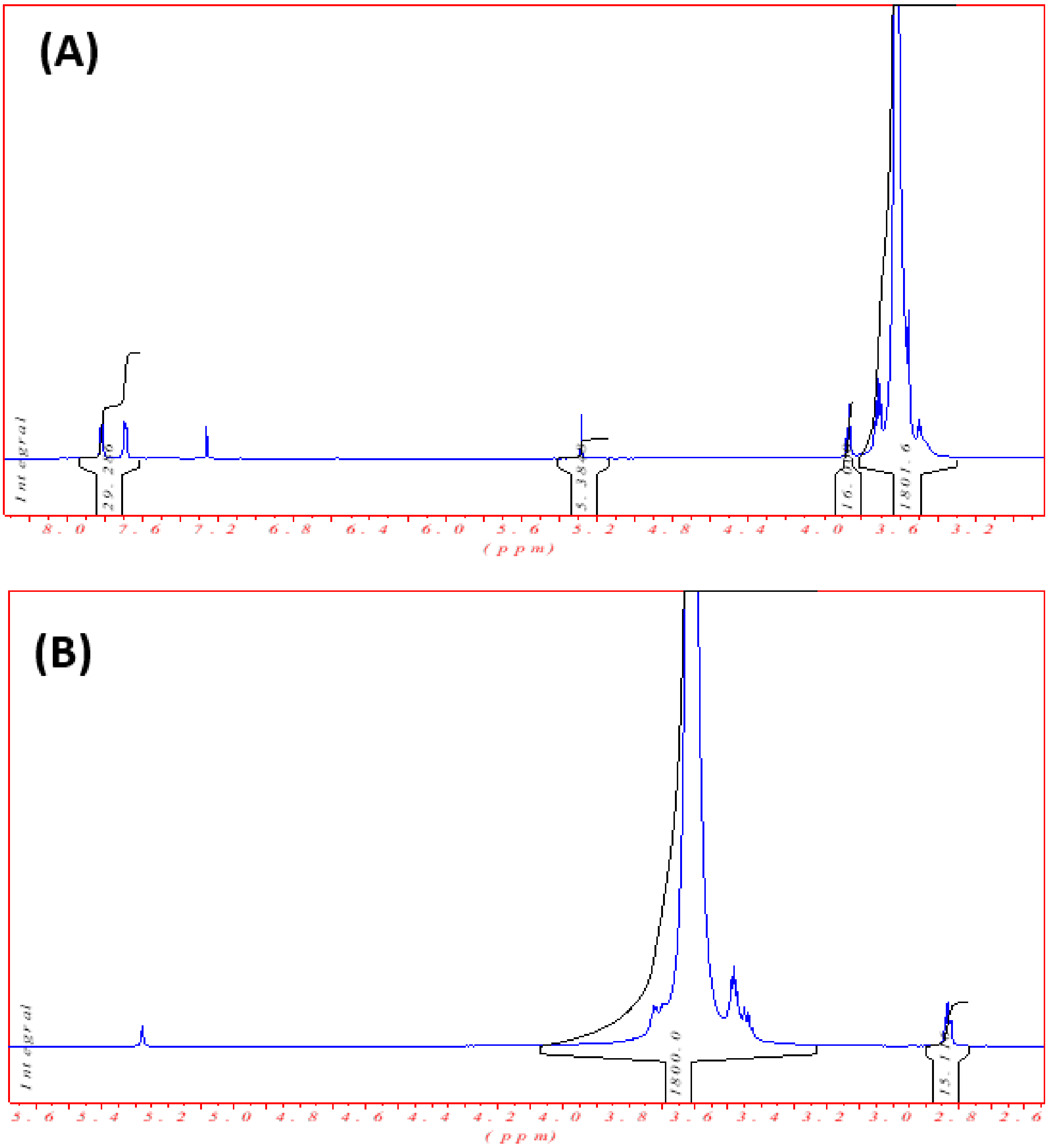

Figure S4. ^{1}H NMR 600 MHz, 8armPEG20k-Phthalimide (A) and 8armPED20k-Amine (B).

5. SI. 5_Figure S5 (A and B)

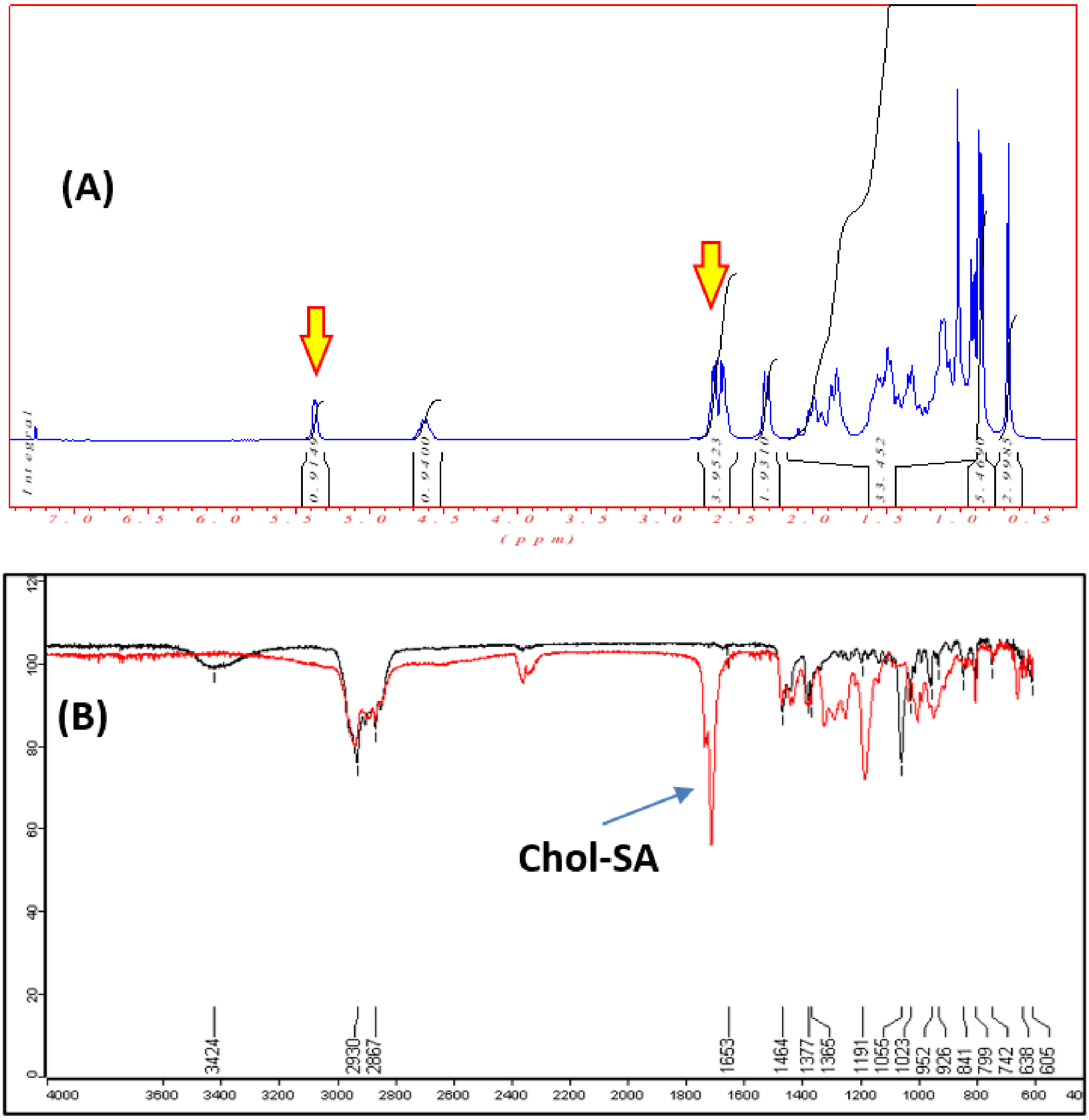

Figure S5. ^{1}H NMR ($CDCl_3$, 300 MHz) (A); IR spectroscopy (B) analysis of cholesterol-propionic acid, compared with cholesterol.

6. SI. 6._Figure S6 and Table S1

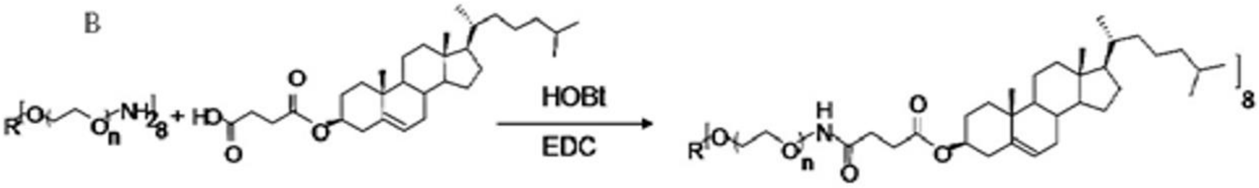

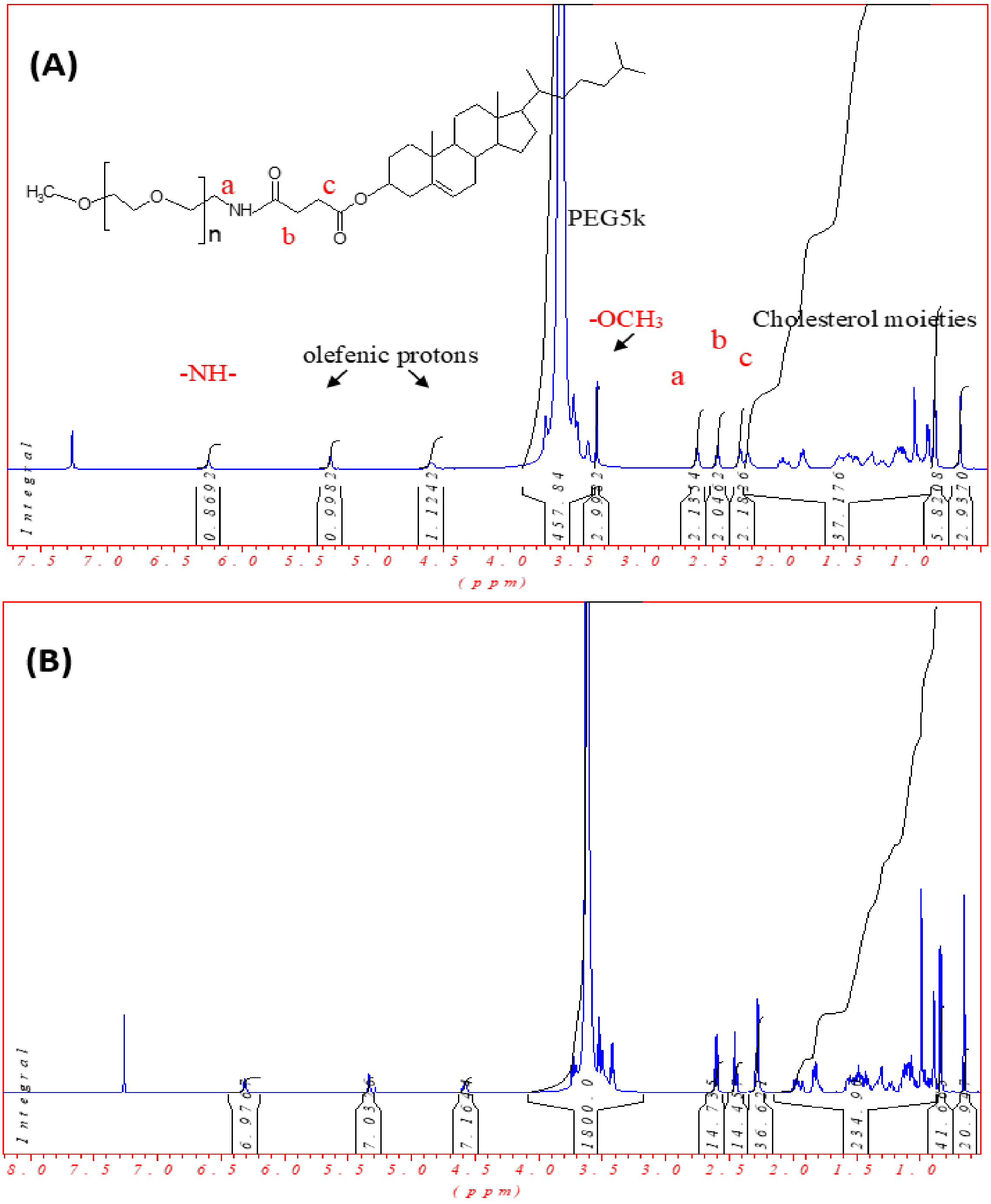

Figure S6. ^{1}H NMR spectra, 600 MHz of mPEG5k-Chol (A) and 8armPEG20k-Chol (B) in $CDCl_3$.

Table S1: Quantification of the NMR signals of the modified PEG-(chol)$_n$ polymers**.**

**Polymer, ppm**		**2.3-2.5 ppm**	**2.65 pmm**	**3.35 pmm**	**3.4 - 4.9 Pmm Reference peaks**	**4.6 pmm**	**5.4 pmm**	**6.3 pmm**	**DS**	**Mwt**
**mPEG5k-Chol**	**Calculated**	4	2	3	458	1	1	1	100	100
**mPEG5k-Chol**	**Collected**	4.1	2.1	2.99	**457.8**	1.1	**0.99**	0.86		99
**8armPEG20k-(Chol)$_8$**	**Calculated**	32	16		1800	8	8	8	100	100
**8armPEG20k-(Chol)7**	**Collected**	28	14		1800	7	**7**	7	7	87.5

7. SI. 7_Figure S7 (A and B)

A)

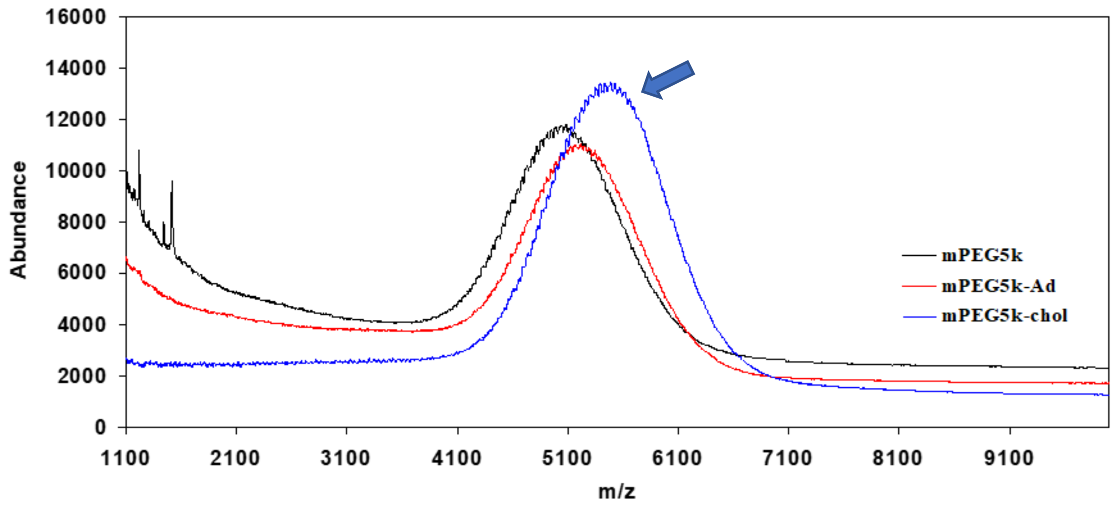

B)

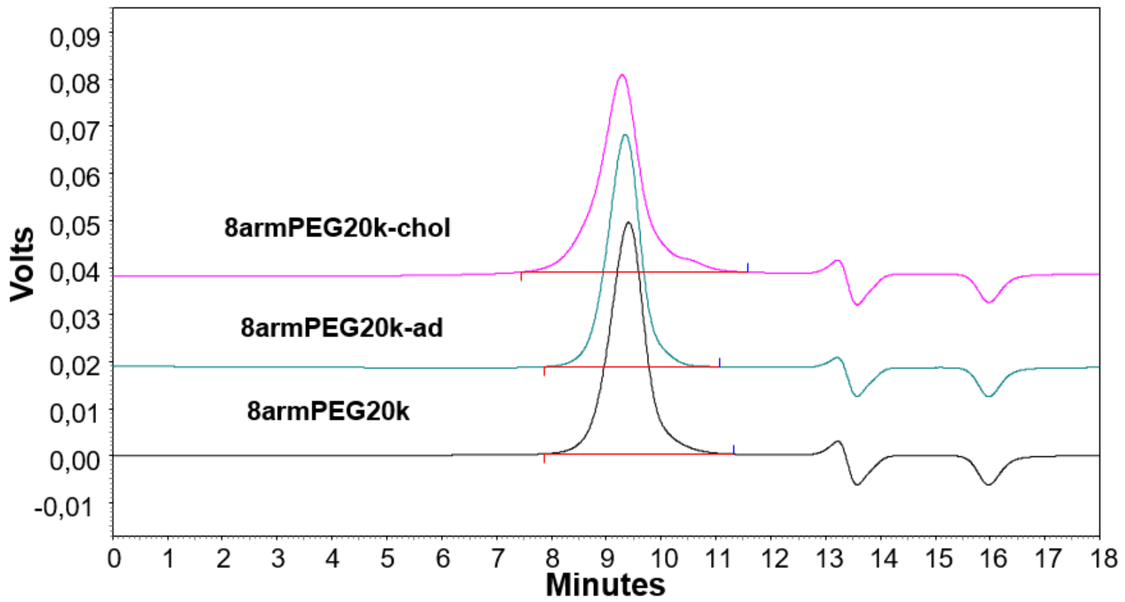

Figure S7. (A) MALDI-ToF mass spectra of unmodified mPEG5k, mPEG5k-Ad and mPEG5k-Chol in THF. The matrix is IAA-THF layer by layer technique. **(B)** GPC profiles of chromatograms of unmodified and hydrophobically modified 8armPEG20k.

8. SI. 8_Figure S8

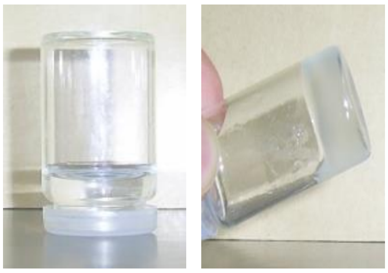

(A) (B)

Figure S8. Fotos of the modified hydrogel, composed of 10% w/v 8armPEG20k-chol/pβ-CD **(B)** in comparison with viscous liquid composed 10% w/v 8armPEG20k-OH/pβ -CD **(A).**

9. SI. 9_Figure S9

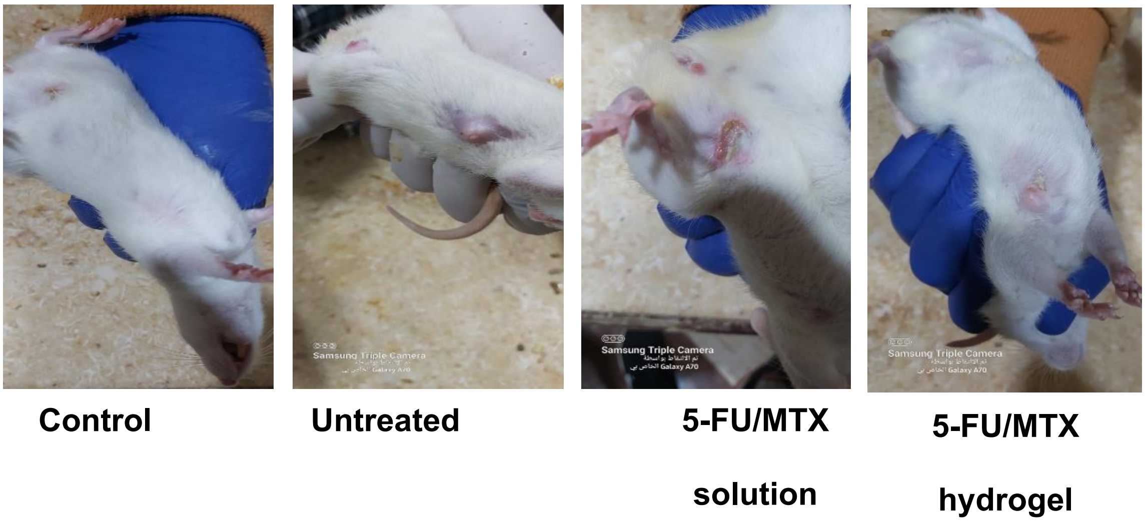

Figure S9. Pictures of the rats bearing breast cancer before and after treatment by 5-FU/ MTX either a saline solution or loaded hydrogel.
